# Genomic and phylogenetic characterisation of an imported case of SARS-CoV-2 in Amazonas State, Brazil

**DOI:** 10.1590/0074-02760200310

**Published:** 2020-09-25

**Authors:** Valdinete Alves do Nascimento, André de Lima Guerra Corado, Fernanda Oliveira do Nascimento, Ágatha Kelly Araújo da Costa, Debora Camila Gomes Duarte, Sérgio Luiz Bessa Luz, Luciana Mara Fé Gonçalves, Michele Silva de Jesus, Cristiano Fernandes da Costa, Edson Delatorre, Felipe Gomes Naveca

**Affiliations:** 1 Fundação Oswaldo Cruz-Fiocruz, Instituto Leônidas e Maria Deane, Manaus, AM, Brasil; 2 Fundação Oswaldo Cruz-Fiocruz, Instituto Oswaldo Cruz, Programa de Pós-Graduação em Biologia Celular e Molecular, Rio de Janeiro, RJ, Brasil; 3 Fundação Oswaldo Cruz-Fiocruz, Instituto Leônidas e Maria Deane, Programa de Pós-Graduação em Biologia da Interação Patógeno-Hospedeiro, Manaus, AM, Brasil; 4 Fundação de Vigilância em Saúde do Amazonas, Manaus, AM, Brasil; 5 Universidade Federal do Espírito Santo, Centro de Ciências Exatas, Naturais e da Saúde, Departamento de Biologia, Vitória, ES, Brasil; 6 Rede Genômica de Vigilância em Saúde do Estado do Amazonas, Manaus, AM, Brasil

**Keywords:** coronavirus, SARS-CoV-2, COVID-19, Brazil, Amazon Region, genome

## Abstract

A new coronavirus [severe acute respiratory syndrome coronavirus 2 (SARS-CoV-2)] is currently causing a life-threatening pandemic. In this study, we report the complete genome sequencing and genetic characterisation of a SARS-CoV-2 detected in Manaus, Amazonas, Brazil, and the protocol we designed to generate high-quality SARS-CoV-2 full genome data. The isolate was obtained from an asymptomatic carrier returning from Madrid, Spain. Nucleotide sequence analysis showed a total of nine mutations in comparison with the original human case in Wuhan, China, and support this case as belonging to the recently proposed lineage A.2. Phylogeographic analysis further confirmed the likely European origin of this case. To our knowledge, this is the first SARS-CoV-2 genome obtained from the North Brazilian Region. We believe that the information generated in this study may contribute to the ongoing efforts toward the SARS-CoV-2 emergence.

The coronavirus disease 2019 (COVID-19) is caused by infection with the severe acute respiratory syndrome coronavirus 2 (SARS-CoV-2) that was identified for the first time in patients with pneumonia in Wuhan, China.^1^ ^,^ ^2^ Since its discovery at the end of 2019, SARS-CoV-2 transmission has been documented as being person-to-person, causing from an asymptomatic status, that may also generate transmission clusters, to a symptomatic disease with the typical symptoms manifested as fever, dry cough, myalgia, fatigue, dyspnea, diarrhea, and nausea.^3^ ^,^ ^4^ In the majority of the patients, the clinical outcome is a mild disease, although Wu and McGoogan described Chinese patients that developed a severe outcome (16%) or a critical condition (4%). Severe or critical outcomes usually occur in patients with comorbidities, and the disease can progress, presenting arrhythmia and shock, that could evolve to death.^5^ ^,^ ^6^

The SARS-CoV-2 belongs to family *Coronaviridae*, genus *Betacoronavirus*. In 2002 and 2012, two outbreaks occurred caused by new coronaviruses, SARS-CoV and MERS-CoV, with lethality ranging from nine to 33%, respectively.^7^ ^,^ ^8^ ^,^ ^9^ The coronaviruses are enveloped viruses, with 80 to 120 nm in diameter. Three proteins compose the virion surface: spike (S), membrane (M), and small membrane protein (E), giving the virus a crown-like visual on electron micrograph.^10^ ^,^ ^11^ This viral family has one of the largest RNA genome of all other RNA viruses, with a single non-segmented positive-stranded RNA of approximately 30 kb.^12^

In Brazil, the first recorded case of SARS-CoV-2 infection occurred in the end of February, in the city of São Paulo, followed by cases in the Northeast (Bahia), Central-West (Brasília) and South (Rio Grande do Sul) regions.^13^ The northern of Brazil was the last region of the country to detected SARS-CoV-2 in its population. In March 13th, the first case in the State of Amazonas was detected in a woman that traveled to England and return to the Amazonas capital, Manaus.^14^ After three days, the second case of SARS-CoV-2 was detected in Manaus and characterised in the present study.

One 56-years-old man returning from Madrid, Spain, arrived asymptomatic in Manaus, Amazonas State, Brazil on 15-Mar-2020. At that time, a massive outbreak of COVID-19 was already established in several European countries, suggesting that travelers should be kept in quarantine at arrival. Even without the symptoms of a respiratory infection, nasal and oropharyngeal swabs were collected for SARS-CoV-2 testing as a routine established for respiratory virus surveillance at Instituto Leônidas e Maria Deane (ILMD) - Fiocruz, Amazonas State, Brazil, since 2019. The swabs were combined and submitted to total nucleic acid extraction with a commercial kit (Biogene, Recife, PE, Brazil) and immediately evaluated using the reverse transcription real-time polymerase chain reaction (RT-qPCR) protocol developed by the US Centers for Disease Control and Prevention (CDC/USA) (On 15-Mar-2020, CDC updated the RT-qPCR protocol removing the N3 target). This RT-qPCR assay employs different primers and probes sets aiming three regions of the SARS-CoV-2 nucleocapsid (N) gene (https://www.fda.gov/media/134922/download), and the human RNase P as an internal control.

The analysed sample tested positive for SARS-CoV-2 with Cts values of 14.43 (N1), 15.39 (N2) and15.33 (N3). Thus, we immediately generated cDNA with random primers and Superscript IV reverse transcriptase (ThermoFisher Scientific, Waltham, MA, United States). We had previously designed a PCR scheme to amplify the entire genome of the SARS-CoV-2 based on an alignment of all complete genome sequences available on GenBank at 03/03/2020. Conserved regions were chosen for primer design with Primer3 v2.3.7. embedded in Geneious Software 10.2.6,^15^ spanning around 2.2Kb and with an overlap region between 131 and 225 bp. This PCR scheme resulted in 15 amplicons with further details presented in the Supplementary data (Table I).

The SARS-CoV-2 whole genome was amplified with Platinum SuperFi II Green PCR master mix (ThermoFisher Scientific) using the 15 primers sets in individual reactions. Each amplicon was then visualised as a unique and intense DNA fragment on agarose gel electrophoresis stained with GelRed (Biotium, Hayward, CA, United States). The PCR amplicons were precipitated with molecular biology grade Polietilenoglicol (PEG) 8,000 (Promega, Madison, WI, United States) and then resuspended in nuclease-free water. After one-hour incubation at 37ºC, all amplicons were quantified in ng/μL using Qubit 2.0 and the dsDNA HS assay kit (Thermo Fisher Scientific). Finally, the number of DNA copies in each purified amplicon was estimated with ENDMEMO (http://www.endmemo.com/bio/dnacopynum.php), normalised, and pooled. A single library was constructed using the Nextera DNA Flex Library Prep and clustered with MiSeq Reagent Micro Kit v2 (300-cycles), following the manufacturer’s protocols. Nucleotide sequencing was performed in the MiSeq platform (Illumina, San Diego, CA, United States), installed at ILMD, in a paired-end run (2x150 cycles).

A total of 10,946,898 reads were trimmed for quality and adapters using BBDUK v37.25, embedded in Geneious software. Thus, 8,362,418 reads were mapped to the SARS-CoV-2 NCBI Reference Sequence NC_045512.2 using Geneious map-to-reference tool. The BR_AM_ILMD_20140001 final consensus genome sequence contains 29,789 nucleotides, with no gaps, a Q40 score of 100%, with no undetermined “N” bases and high average coverage (> 34,000X). To avoid any primers bias, we removed both primer binding sites at the 5′ and 3′ ends. Thus, our final sequence represents the positions between nucleotides 47 and 29,835 or 99.6% of the NCBI RefSeq previously mentioned.

We aligned the BR_AM_ILMD_20140001 genomic sequence with the SARS-CoV-2 NCBI Reference Sequence NC_045512 using MAFFT v7.388^16^ to investigate any mutations throughout genome. A total of nine mutations were observed at nucleotide positions 8,782 (C to T); 9,477 (T to A); 12,781 (C to T); 14,805 (C to T); 25,979 (G to T); 26,642 (C to T); 28,144 (T to C); 28,657 (C to T) and 28,863 (C to T), with four of these leading to residues substitution in the deduced protein sequences (Table).

**TABLE:** Differences observed between sample BR_AM_ILMD_20140001 and the severe acute respiratory syndrome coronavirus 2 (SARS-CoV-2) prototype

Genome position	8782	9477	12781	14805	25979	26642	28144	28657	28863
NC_045512	C	T	C	C	G	C	T	C	C
BR_AM_ILMD	T	A	T	T	T	T	C	T	T
Codon position	AG**C**	TTT	TA**C**	TA**C**	GGA	GCC	TTA	GA**C**	T**C**A
	AG**T**	T**A**T	TA**T**	TA**T**	GTA	GCT	T**C**A	GA**T**	TTA
Mutation type	s	ns	s	s	ns	s	ns	s	ns
Protein	nsp4	nsp4	nsp9	RdRp	ORF3a ptn	M glycoptn	ORF8 ptn	npptn	npptn
Residue	Ser2839	Phe3071Tyr	Tyr4172	Tyr4847	Gly196Val	Ala40	Leu84Ser	Asp128	Ser197Leu

Nucleotides substituted in each codon are represented in bold. s: silent mutation; ns: non-silent mutation; nsp: non-structural protein; RdRp: RNA-dependent RNA-polymerase; ORF3a ptn: ORF3a protein; M glycoptn: M glycoprotein; ORF8 ptn: ORF8 protein; npptn: nucleocapsid phosphoprotein.

To put the BR_AM_ILMD_20140001 genome in a global context, we aligned the new genome to a pool of all SARS-CoV-2 genomes with at least 25,000 nucleotides available at GISAID database^17^ on March 31st, 2020 using MAFFT. We adopted a subsampling strategy to reduce computation time, selecting subsets of 15-20 sequences retaining the most viral diversity from each country using the CD-HIT program.^18^ This subsampling approach resulted in a final sequence dataset with 490 sequences from 53 countries [Supplementary data (Table II)].

Complete coding sequences (CDS) were subjected to maximum likelihood (ML) phylogenetic reconstruction with PhyML v3.0,^19^ under the HKY+Γ4 nucleotide substitution model. According to the ML phylogenetic tree, the BR_AM_ILMD_20140001 sequence belonged to the lineage A^20^ (Fig. 1A) clustering within a monophyletic cluster (aLRT = 1.00) comprising sequences from Spain, Chile, France, Greece, Georgia, Netherlands, Senegal (Fig. 1B). The pangolin web application (pangolin.cog-uk.io) further assigned the sequences from this cluster to the A.2 lineage (Fig. 1B).

**Fig. 1:**
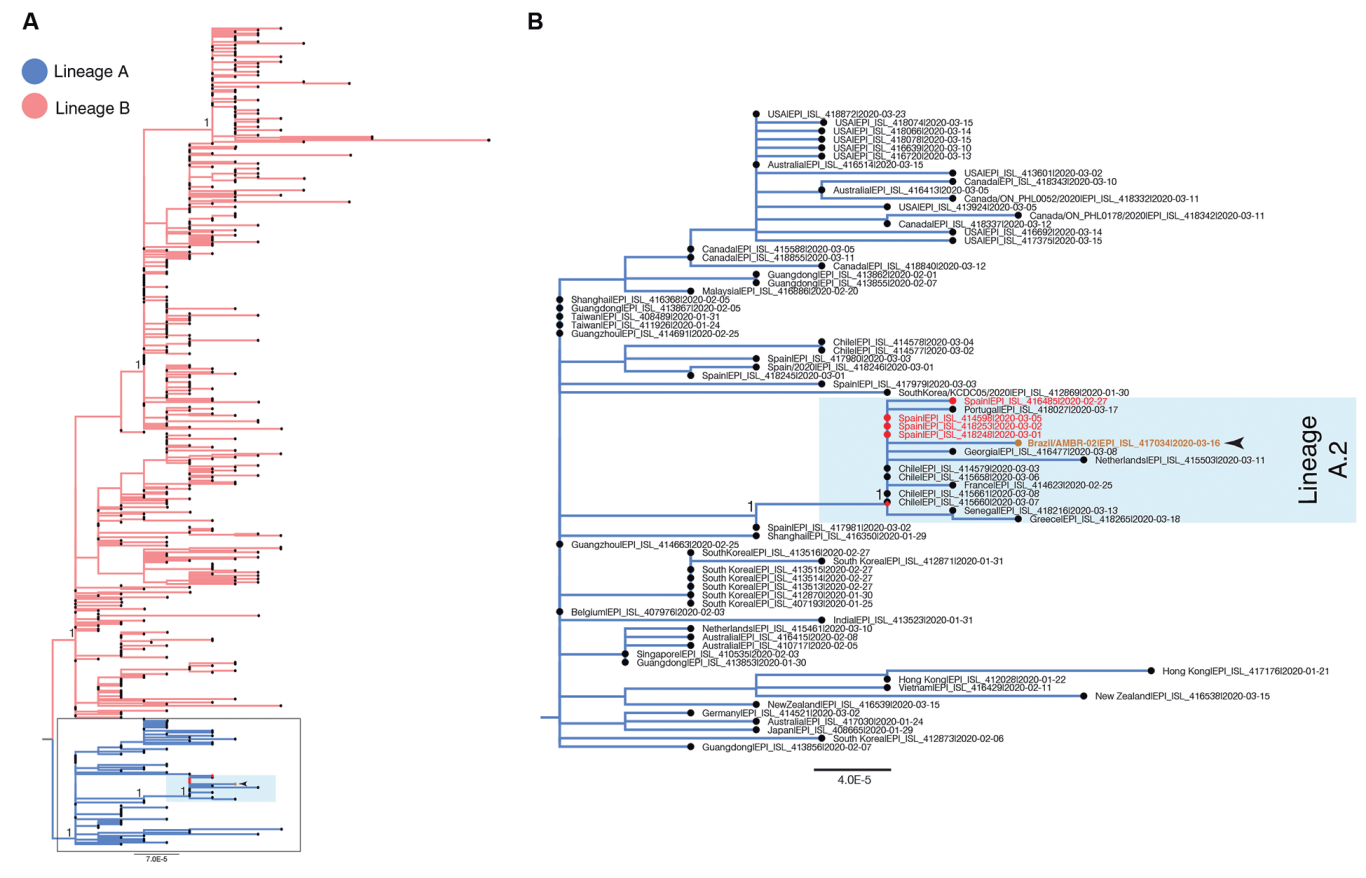
maximum-likelihood (ML) phylogeny of subsampled severe acute respiratory syndrome coronavirus 2 (SARS-CoV-2) genomes. (A) ML tree rooted on the branch separating lineages A and B sequences colored following the legend. (B) A close view of the lineage A highlighting the A.2 lineage (light blue box) that comprises the BR_AM_ILMD_20140001 strain (indicated with an arrow). The nodes representing the most recent common ancestor (MRCA) of the lineage A.2 is indicated with a red diamond. In both trees, tips representing the Spanish strains inside lineage A.2 are coloured red and the scale bar represents nucleotide substitutions per site.

After temporal validation with Tempest^21^ (Fig. 2A), we conducted a Bayesian discrete spatiotemporal analysis with all lineage A sequences (Fig. 2) using the BEAST v1.10 package,^22^ applying the strict molecular clock and the parametric exponential coalescent models. The strict molecular clock model was selected over the relaxed uncorrelated one,^23^ after marginal likelihood estimation using path sampling and stepping-stone sampling methods^24^ [Supplementary data (Table III)].The analysis under the uncorrelated relaxed molecular clock model, however, resulted in estimates similar to those of the strict clock model (data not shown). We estimated that the most recent common ancestor (MRCA) of lineage A.2 originated in Spain (posterior state probability, PSP = 0.99) in the beginning of February 2020 (95% highest posterior density, HPD = 2nd - 22nd Feb 2020). The phylogeographic analysis also pointed out that the BR_AM_ILMD_20140001 was most probably introduced from Spain (PSP = 0.43) (Fig. 2B).

**Fig. 2:**
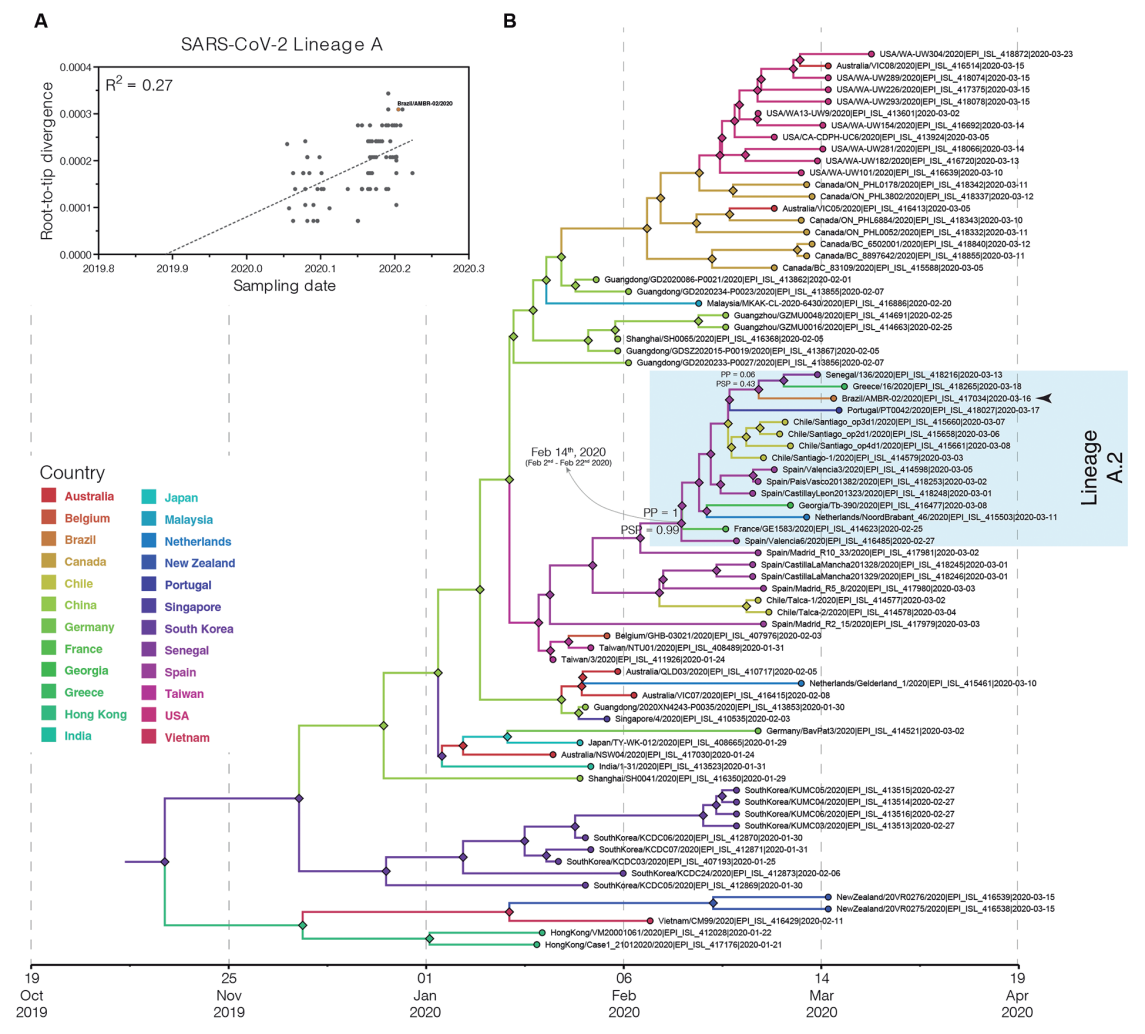
phylogeography of the severe acute respiratory syndrome coronavirus 2 (SARS-CoV-2) lineage A. (A) Temporal signal analysis correlating the sampling date of each sequence and its genetic distance from the root of a maximum likelihood phylogeny. (B) Time-scaled Bayesian phylogeographic maximum clade credibility (MCC) tree of the SARS-CoV-2 complete coding sequences (CDS) classified as lineage A. The branch’s colors represent the most probable location of their descendent nodes (diamonds) as indicated at the legend. Branch support are indicated only at key nodes posterior (PP) and posterior state probability (PSP). The lineage A.2 is highlighted with a light blue box. All horizontal branch lengths are drawn to a scale of years.

Like other viral infections, the infection by SARS-CoV-2 can be asymptomatic and this fact has been reported in different studies.^25^ ^,^ ^26^ ^,^ ^27^ It is noteworthy that according to the definition of suspected case adopted in Brazil at the time that we investigated this asymptomatic carrier, he would not be included in SARS-CoV-2 testing, despite returning from an area with active transmission like Madrid, Spain.^28^ Until 24-Mar-2020, when we deposited the BR_AM_ILMD_20140001 genome information at https://www.gisaid.org, there were only 17 Brazilian SARS-CoV-2 complete genome sequences, and the sequence reported in the present work is the first complete genome from the northern Brazilian region.

Several laboratories over the world are now sequencing thousands of SARS-CoV-2 genomes, which is undoubtedly the most notable effort of viral sequencing in human history. Like any other virus, the new coronavirus is continuously evolving as more hosts, humans or animals, are getting infected. Thus, it is of paramount importance to generate and share high-quality full viral genomes from different regions over the world to better understand the SARS-CoV-2 evolution. The information related to the viral evolution is not only necessary for molecular epidemiology studies, but also to monitor if the newly identified mutations are linked to different clinical presentations or may drive into false-negative results when performing nucleic acid amplification assays, like real-time PCR.

Therefore, in this work, we aimed to describe and characterise the complete genome of the SARS-CoV-2 obtained from an asymptomatic carrier returning from Madrid, Spain. To achieve this goal, we decided to use a nucleotide sequencing strategy where firstly all the 15 amplicons, encompassing the entire SARS-CoV-2 genome, were confirmed by agarose gel electrophoresis. Subsequently, each amplicon was quantified and normalised in order to prevent that one region could be overrepresented during sequencing. In order to make our approach more straightforward, we decided to evaluate if longer amplicons could be generated in a very similar way. We were successful in generating amplicons around 6 Kb, with a minimum overlap of 131 bp, reducing the number of PCR reactions to 5 instead of 15 (data not showed). The conditions to amplify the 6 Kb amplicons followed the manufacturer’s recommendations, with the primers pairs details presented in the Supplementary data (Table I) of this manuscript. Of note, we observed that only samples with high viral load (e.g. Ct lower than 21) were fully amplified using the 6Kb protocol. We believe that this approach may be exciting not only to reduce the current protocol costs, but also for those interested in using long reads sequencing technologies like PacBio SMS and nanopore.

Recently, Rambaut and colleagues proposed a rational and dynamic virus classification for SARS-CoV-2 genomes based on a phylogenetic framework.^20^ Using this approach authors identified at the root of the phylogeny of SARS-CoV-2 two lineages that were simply denoted as lineages A and B. In our analysis, while all Brazilian SARS-CoV-2 sequences belonged to the lineage B, mostly from the B.1 lineage, the BR_AM_ILMD_20140001 genome clustered within the lineage A.2, indicating that BR_AM_ILMD_20140001 strain belongs to a distinct transmission cluster than the other full Brazilian genomes reported until March 31st, 2020. Originally, the lineage B.1 predominated in Europe and North America,^17^ ^,^ ^20^ and currently, constitutes the most prevalent SARS-CoV-2 variant circulating in Brazil.^29^ The lineage A.2 constitutes a predominantly Spanish lineage, found in at least 25 countries^20^ and our phylogeographic analysis corroborates its origins and reinforces the importation scenario from Spain to Brazil.

In this study, we report and characterise the first SARS-CoV-2 genome obtained from an infected subject in the Brazilian North Region. Since this case was an asymptomatic carrier, it is not easy to suggest when infection has occurred. However, our phylogeographic analysis strongly indicates this individual was infected in Spain. Finally, we would like to emphasise that more fully genomes studies of the SARS-CoV-2 are necessary to better understand the evolution of this emerging life-threatening virus and the information of the nucleotide sequence described here may contribute to future molecular epidemiological studies in Brazil. In this sense, the protocol that we described in the present study may be useful to aid other researchers to generate other high-quality SARS-CoV-2 genomes.

*Nucleotide sequence accession number* - The complete genome sequence of the BR_AM_ILMD_20140001 isolate is available in GISAID since March 24, 2020, under the ID number EPI_ISL_417034.
