# Supplementary material for: Genomic and phylogenetic characterisation of an imported case of SARS-CoV-2 in Amazonas State, Brazil
Source: Mem Inst Oswaldo Cruz. 2020 Sep 25;115:e200310. doi: 10.1590/0074-02760200310 (PMC7523504; doi:10.1590/0074-02760200310)
Supplement: Supplementary file 1 [file 1678-8060-mioc-115-e200310-s1.pdf]

TABLE I

Primers for severe acute respiratory syndrome coronavirus 2 (SARS-CoV-2) sequencing designed in this study

Name	Primer Sequence	Primer size (bases)
SARSCOV2_24_FNF	GTAACAAACCAACCAACTTTCGA	23
SARSCOV2_1847_FNF	TCAATACTGAGTCCTCTTTATGCAT	25
SARSCOV2_1978_FNR	ATCTAGTATTGTTATAGCGGCCTTC	25
SARSCOV2_3841_FNF	AATGAAGAGTGAAAAGCAAGTTGAA	25
SARSCOV2_3986_FNR	TTGTAACTTCTTCAACACAAGCTTT	25
SARSCOV2_5830_FNF	GTCCTCAGAATACAAAGGTCCTATT	25
SARSCOV2_5997_FNR	GGTTGCTCTGTGAAATAAGAATTGT	25
SARSCOV2_7836_FNF	TAGACAACCTGAGAGCTAATAACAC	25
SARSCOV2_7966_FNR	TTGACACATAAGCTGACTGTAGTAA	25
SARSCOV2_9845_FNF	CGTAGTGATGTGCTATTACCTCTTA	25
SARSCOV2_10069_FNR	TTTTCTAAAACCACTCTGCAAAACA	25
SARSCOV2_11857_FNF	TGTAAAGTGCACATCAGTAGTCTTA	25
SARSCOV2_11989_FNR	AAAGGCTTCAGTAGTATCTTTAGCT	25
SARSCOV2_13938_FNF	AGAAAACCCAGATATATTACGCGTA	25
SARSCOV2_14098_FNR	CGAAATCATACCAGTTACCATTGAG	25
SARSCOV2_15911_FNF	ATGATTATGTGTACCTTCCTTACCC	25
SARSCOV2_16060_FNR	TAGGATGTTTAGTAAGTGGGTAAGC	25
SARSCOV2_17891_FNF	CAGCTCACTCTTGTAATGTAAACAG	25
SARSCOV2_18058_FNR	GTCCTGTTACATTTTCAGCTTGTAA	25
SARSCOV2_19865_FNF	ATACTGTGATCTGGGACTACAAAAG	25
SARSCOV2_20034_FNR	ACGGGCATTTCTAAATAAGTCTACT	25
SARSCOV2_21821_FNF	AATGATGGTGTTTATTTTGCTTCCA	25
SARSCOV2_22007_FNR	TGTTTTTGTGGTAATAAACACCCAA	25
SARSCOV2_23997_FNF	CAAGCAAGAGGTCATTTATTGAAGA	25
SARSCOV2_24144_FNR	AAAACAGTAAGGCCGTTAAACTTTT	25
SARSCOV2_25827_FNF	TTTTCTTTGCTGGCATACTAATTGT	25
SARSCOV2_26032_FNR	GCTGGTAATAGTCTGAAGTGAAGTA	25
SARSCOV2_28155_FNF	ATTAATTGCCAGGAACCTAAATTGG	25
SARSCOV2_29672_FNR	ATGTGAGATTAAAGTTAACTACATCT	26
SARSCOV2_29861_FNR	CTAAGAAGCTATTAAAATCACATGGG	26
SARSCOV2_W_HU_1_28946_FNR	CAAGCAGCAGCAAAGCAAGA	20

15 amplicons scheme

Amplicon	Forward	Reverse	Bp	Overlap (bp)
1	SARSCOV2_24_FNF	SARSCOV2_1978_FNR	1955	132
2	SARSCOV2_1847_FNF	SARSCOV2_3986_FNR	2140	146
3	SARSCOV2_3841_FNF	SARSCOV2_5997_FNR	2157	168
4	SARSCOV2_5830_FNF	SARSCOV2_7966_FNR	2137	131
5	SARSCOV2_7836_FNF	SARSCOV2_10069_FNR	2234	225
6	SARSCOV2_9845_FNF	SARSCOV2_11989_FNR	2145	133
7	SARSCOV2_11857_FNF	SARSCOV2_14098_FNR	2242	161
8	SARSCOV2_13938_FNF	SARSCOV2_16060_FNR	2123	150
9	SARSCOV2_15911_FNF	SARSCOV2_18058_FNR	2148	168
10	SARSCOV2_17891_FNF	SARSCOV2_20034_FNR	2144	170
11	SARSCOV2_19865_FNF	SARSCOV2_22007_FNR	2143	187
12	SARSCOV2_21821_FNF	SARSCOV2_24144_FNR	2324	148
13	SARSCOV2_23997_FNF	SARSCOV2_26032_FNR	2036	206
14	SARSCOV2_25827_FNF	SARSCOV2_W_HU_1_28946_FNR	3120	792
15	SARSCOV2_28155_FNF	SARSCOV2_29861_FNR	1707	

5 amplicons scheme

Amplicon	Forward	Reverse	Bp	Overlap (bp)
1	SARSCOV2_24_FNF	SARSCOV2_5997_FNR	5974	168
2	SARSCOV2_5830_FNF	SARSCOV2_11989_FNR	6160	133
3	SARSCOV2_11857_FNF	SARSCOV2_18058_FNR	6202	168
4	SARSCOV2_17891_FNF	SARSCOV2_24144_FNR	6254	148
5	SARSCOV2_23997_FNF	SARSCOV2_29861_FNR	5865	

TABLE II

Sequence dataset used in this study after subsampling strategy

Country	GISAIDdataset (n)	Subsampled dataset (n)
Algeria	2	2
Australia	65	19
Belgium	88	18
Brazil	16	16
Cambodia	1	1
Canada	106	19
Chile	7	7
China	211	19
Congo	12	12
CzechRepublic	3	3
Denmark	9	9
Finland	32	19
France	108	17
Georgia	10	10
Germany	21	21
Greece	3	3
HongKong	20	20
Hungary	3	3
Iceland	269	16
India	1	1
Ireland	10	10
Italy	18	18
Japan	79	18
Kuwait	4	4
Lithuania	1	1
Luxembourg	10	10
Malaysia	7	7
Mexico	1	1
Nepal	1	1
Netherlands	118	17
New Zealand	4	4
Norway	6	6
Pakistan	1	1
Panama	1	1
Peru	1	1
Poland	1	1
Portugal	42	14
Russia	1	1
Saudi Arabia	3	3
Scotland	5	5
Senegal	10	10
Singapore	11	11
Slovakia	3	3
SouthAfrica	1	1
SouthKorea	13	13
Spain	31	18
Sweden	1	1
Switzerland	30	17
Taiwan	17	16
Thailand	2	2
United Kingdom	167	16
USA	622	18
Vietnam	5	5
Total	2214	490

TABLE III

Best fit molecular clock model for the severe acute respiratory coronavirus 2 (SARS-CoV-2) A.2 dataset Bayesian phylogeographic analysis

Marginal likelihood estimation	Clock model		Bayes Factor (Strict/UCLD)
	Strict	UCLN	
Path sampling	-41604.7	-41615.1	10.4
Stepping-stone	-41604.9	-41615.4	10.5

Log marginal likelihood (ML) estimates for strict clock and uncorrelated relaxed clock with a lognormal distribution (UCLD) models obtained using the path sampling (PS) and stepping-stone sampling (SS) methods. The Log Bayes factor (BF) is the difference of the Log ML between of alternative (H1) and null (H0) models (H1/H0). Log positive BFs values indicates that model H1 is more strongly supported by the data than model H0, while negative values indicate that BF is in favor of the model H0. Each model was systematically compared, and higher BF values were highlighted in bold while the best fit model was underlined.

TABLE IV
click here to access
